# Advancing towards Ubiquitous EEG, Correlation of In-Ear EEG with Forehead EEG

**DOI:** 10.3390/s22041568

**Published:** 2022-02-17

**Authors:** Swati Mandekar, Abigail Holland, Moritz Thielen, Mehdi Behbahani, Mark Melnykowycz

**Affiliations:** 1 Institute for Bioengineering, University of Applied Sciences Aachen, 52005 Aachen, Germany; swati.mandekar@alumni.fh-aachen.de (S.M.); behbahani@fh-aachen.de (M.B.); 2 IDUN Technologies AG, Alpenstrasse 3, 8152 Glattpark, Switzerland; abigailholland22@gmail.com (A.H.); moritz@iduntechnologies.ch (M.T.)

**Keywords:** in-ear EEG, correlation, forehead EEG, impedance spectroscopy, biopotential electrodes, BCI

## Abstract

Wearable EEG has gained popularity in recent years driven by promising uses outside of clinics and research. The ubiquitous application of continuous EEG requires unobtrusive form-factors that are easily acceptable by the end-users. In this progression, wearable EEG systems have been moving from full scalp to forehead and recently to the ear. The aim of this study is to demonstrate that emerging ear-EEG provides similar impedance and signal properties as established forehead EEG. EEG data using eyes-open and closed alpha paradigm were acquired from ten healthy subjects using generic earpieces fitted with three custom-made electrodes and a forehead electrode (at Fpx) after impedance analysis. Inter-subject variability in in-ear electrode impedance ranged from 20 kΩ to 25 kΩ at 10 Hz. Signal quality was comparable with an SNR of 6 for in-ear and 8 for forehead electrodes. Alpha attenuation was significant during the eyes-open condition in all in-ear electrodes, and it followed the structure of power spectral density plots of forehead electrodes, with the Pearson correlation coefficient of 0.92 between in-ear locations ELE (Left Ear Superior) and ERE (Right Ear Superior) and forehead locations, Fp1 and Fp2, respectively. The results indicate that in-ear EEG is an unobtrusive alternative in terms of impedance, signal properties and information content to established forehead EEG.

## 1. Introduction

A number of imaging techniques have been developed over recent decades to provide more knowledge on the anatomy and function of the brain, such as Electroencephalography (EEG), Positron emission tomography (PET), Magnetic resonance imaging (MRI), and Functional near infrared spectroscopy (fNIRS). However, many neuroimaging techniques are not well-suited for mobile and lifestyle integration where the imaging technique may be used throughout the day to understand how the brain is changing due to different environmental stimuli or as connected to specific brain disorders and treatments a person may be undertaking. EEG offers great promise as a brain imaging technique that may integrate as a lifestyle product, similar to the way that smart watches may be used to capture data on the movement and cardiac activity of a person. One promising way to measure EEG ubiquitously and conveniently is to measure signals from the ear canal, and thereby functionalize common earbud products with brain-computer interface (BCI) capabilities.

Biopotential measurements can be used as a diagnostic tool providing healthcare professionals with unique insights to detect diseases and take preventive measures accordingly. EEG, electromyography (EMG), and electrocardiography (ECG) are examples of non-invasive diagnostic procedures. EEG is a popular brain imaging technique used by scientists and physicians. Groups of neurons transmit ionic currents, which create individual voltage fluctuations. EEG can leverage the propagation of these voltage signals to measure electrical potentials at the scalp as a method of approximating brain activity. Among various biopotential recordings, EEG has received significant attention in the medical and consumer device market in recent years. EEG is a versatile tool for extracting a wide range of information. Widespread applications of EEG devices include sleep monitoring [1] as well as brain and cognitive health tracking for mental health [2]. By 2026, the global market for EEG devices is projected to reach US $8.85 billion [3]. Factors such as the increasing prevalence of neurovascular diseases, health awareness among individuals, and a rapidly expanding aging population accelerate the market growth [4]. Most of the current application is limited to clinics and research as EEG systems are typically bulky and require trained professionals to operate correctly.

Traditionally used clinical EEG systems require a conductive gel to form a stable, conductive pathway from the scalp to the electrodes. Additionally, the outermost skin layer, the stratum corneum, is abraded to remove dead skin cells to achieve a low skin-contact impedance. This can cause skin irritation and discomfort and results in decreased signal quality over time as the gel dries [5]. In recent years, the consumer market has been growing and effectively addressing many of these constraints. Personal brain metrics monitoring has gained significant traction in the wearable market because consumers, companies and institutions can leverage this technology for everyday health monitoring. Numerous companies are developing consumer-friendly solutions for specific personalized applications through neurofeedback. These low-cost devices use a limited number of electrodes and are used for preventive health monitoring applications, such as meditation [6], sleep tracking [7], headache prevention [8], and fatigue management [9]. Table 1 below highlights some of the current low-cost EEG devices, their electrode configurations, electrode placements according to the International 10–20 electrode placement scheme (The International 10–20 system is an internationally recognized system for identifying scalp regions for electrode placement during EEG measurement.) and their intended applications.

These devices are mobile, noninvasive, and mainly utilize electrode locations on the forehead as the hair-free electrode-skin contact enables improved signal quality. Headbands and other current systems can be uncomfortable to wear for extended periods of time and are inconvenient to use while sleeping. Furthermore, if the user does not place them correctly, data acquisition can become disrupted. To improve the usability of EEG systems, it can be beneficial to move the electrode arrangement away from the forehead to a more convenient and unobtrusive location.

To tackle all these problems, Ear-EEG has emerged as a promising alternative in recent years. Ear-EEG is a novel technique that uses earpieces to record EEG from the ear canal and concha, and around the ear [10]. It makes continuous biopotential monitoring ubiquitous as it can be worn in everyday life just like earphones. Moreover, it makes long-term brain recording more discreet, user-friendly, and unobtrusive [11,12]. Compared to the scalp, it utilizes a lower number of electrodes located in close proximity to the temporal regions, yet signal characteristics are comparable to the scalp EEG for specific paradigms [10,13,14]. Applications relying on high temporal resolution include sleep monitoring [15], epilepsy seizure detection [16], stress management [2] and meditation [17]. For medical applications such as epilepsy, the epileptiform abnormalities can be interpreted from EEG, but the localization of these in the brain still needs to be investigated [18].

Hans Berger, the first person to successfully capture EEG data from a human being, observed that EEG oscillations in the alpha band (8–13 Hz) decreased in amplitude or disappeared completely when observers opened their eyes. This effect is known as the “Berger effect” or “alpha blocking”, named after Berger and the phenomenon [19]. This alpha blocking implies the desynchronization of oscillators in the brain after the opening of eyes, whereas alpha waves are an indicator of the synchronized activity in the brain [19]. Numerous research has used alpha attenuation as a paradigm for validating the proper functioning of EEG recording equipment due to its robust nature and its ability to be evoked in most individuals [11,20,21]. Apart from validating EEG recording equipment, the alpha paradigm has also been used for the detection of autism [22], stress monitoring [23], functional mechanism of selective attention [24], and in meditation studies [17]. Alpha-band oscillations are believed to be associated with many fundamental cognitive processes [25]. This research uses the alpha paradigm to compare EEG recordings acquired from the forehead and ear, as well as to verify the system’s validity. In this investigation, we do not try to localize alpha generators, as attempts to localize alpha-activity generators have been addressed in the past [26].

Many brain regions are active during cognitive processes, including the frontal, parietal, temporal, and occipital. So, the observed brain activity measured at one surface location is an overall result of activity from many sub-regions. D. Looney et al. proved high coherence between temporal, T7, and mastoid, M1 (International 10–20 electrode system), electrodes on the scalp with an ear canal electrode, implying the shared activity between the scalp and ear electrodes [11]. The T7 position is located above the left ear, while M1 is the left mastoid. This established that activity from the scalp is measurable from the ear; however, no attempt was made to investigate the relationship between activity from the frontal and in-ear regions. Therefore, the purpose of this study is to examine this relationship by calculating the alpha band power correlation between those locations. In the process, the optimal position of the in-ear electrodes is investigated. Following a preliminary evaluation of the electrode-skin impedance, EEG measurements were performed using in-ear electrodes in parallel with forehead locations. The impedance values were expected to be in few kΩ (10 to 50 kΩ), and the Berger effect was expected to be present at both locations, which is the attenuation of alpha during the eyes-open condition compared to eyes-closed. The quality of the recorded signals was estimated by evaluation of the signal-to-noise ratio (SNR). Different features on EEG data were computed to compare the forehead electrodes with the in-ear electrodes.

The current study contributes to our understanding of the correlation of forehead to the in-ear electrodes as well as the impact of the placement of electrodes on signal pickup. By demonstrating that the EEG activity recorded on the forehead may also be measurable at the outer ear canal, new applications resulting in an unobtrusive and user-friendly experience for in-ear EEG technology can be established in the future.

## 2. Materials and Methods

### 2.1. Earpiece Production and Preparation

**Figure 1:**
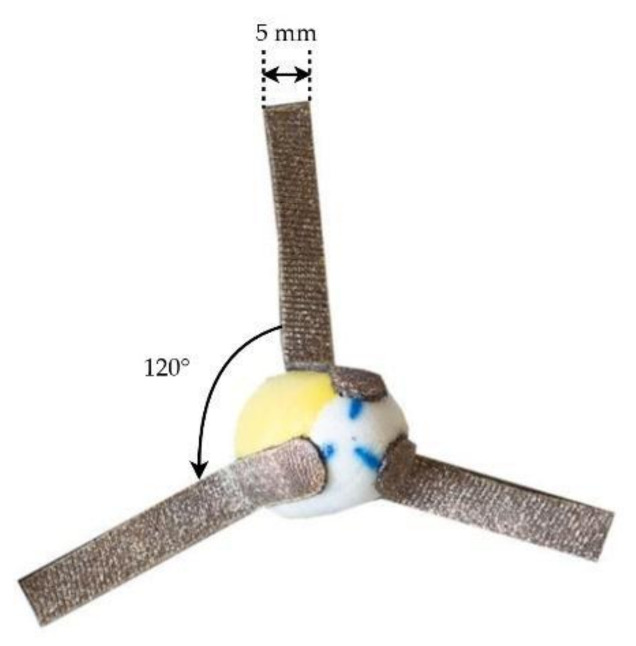
Foam earplug earpieces with three electrodes located at 120° apart of sizes 8 × 5 mm (area: 40 mm^2^).

A memory foam substrate is used for the production of in-ear electrodes. Memory foam has the ability to withstand severe compression without damage, which is necessary for comfortable and easy insertion. The pressure on the external surface of the earplug is uniformly distributed, resulting in great contact with the skin at any location on the earpiece, resulting in a durable electrode-skin interface and comfort over time. Hence, a memory foam earplug was used as the earpiece structure to provide more flexibility for long-duration monitoring and adaptability to the unique ear canal geometries of test participants. To withstand the high level of compression applied to foam earplugs before insertion, the electrodes must be equally flexible yet robust. Electrodes were fabricated from silver-plated knitted conductive fabric (surface resistivity < 5 Ω/sq) attached to the memory foam core. Stretchable fabric quickly accommodates all required deformations, maintains a low impedance and is simple to attach to the substrate and wires. The rectangular form of the electrodes (5 mm × 8 mm) was manufactured using a laser cutter. Electrodes were adhered to the surface of the foam earplugs using a biocompatible adhesive, which allowed them to retain their structural integrity following application. Three electrodes, each having a total surface area of 40 mm^2^, were positioned 120° apart and equidistant from one another (as shown in Figure 1). The edges of the electrodes were slightly rounded to improve the comfort of the test subjects.

Wires were crimped to the electrodes and a pin was used for the connectors. Medical adhesive tape was used to ensure separation between each electrode, limiting the likelihood of linking. Twenty total earpieces were used to upkeep proper hygiene and allow for one earpiece set to be used per subject.

### 2.2. Participants

Ten young and healthy individuals participated in the study (mean age 28 ± 2.4, 5 males and 5 females). The ear canal sizes of the participants varied from small (5 participants), medium (3 participants) to large (2 participants). An equal number of males and females were recruited to keep the findings unbiased with regards to gender. All participants signed a written informed consent form in accordance with the Declaration of Helsinki prior to the experiments.

### 2.3. Electrode Placement Nomenclature

**Figure 2:**
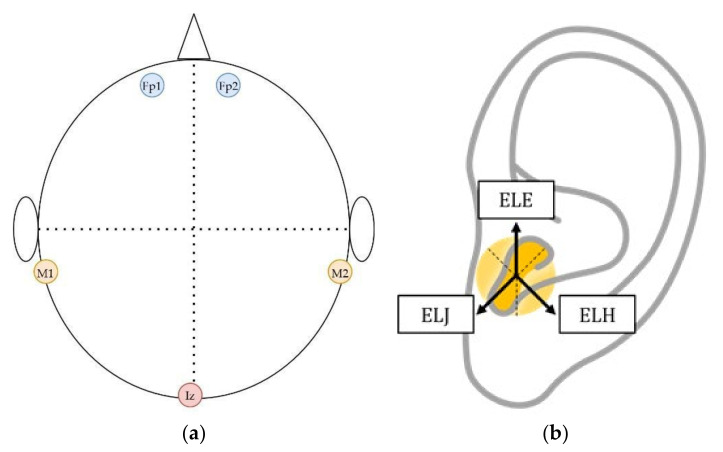
(**a**) Electrode placement on the head for EEG measurement according to 10–20 International electrode placement, Fp1 and Fp2 refer to forehead locations, M1 and M2 are mastoids and Iz is inion; (**b**) labeling scheme for the in-ear electrodes, Exy where x ∈ {L, R} refers to the left or right ear and y is the position of the electrodes.

The labeling scheme for the in-ear electrode locations was based on P. Kidmose et al. [17] and was used throughout the experiment. The electrodes are labeled by Exy, where x ∈ {L, R} refers to electrodes in the left or right ear, and y refers to the electrode placement in the ear canal, respectively. The electrode locations for earpieces selected in this study were E, J, and H. The earpieces were inserted in the subject’s ear, such that the three electrodes on the earpieces made contact at the E, J, and H positions, as shown in Figure 2 (electrode location drawing redone from Kappel [21]). The forehead locations used were Fp1 and Fp2, according to the International 10–20 electrode placement system. The associated in-ear and forehead positions were determined by hemisphere, with the right in-ear EEG system associated with the right frontal position with a similar trend on the left hemisphere.

**Figure 3:**
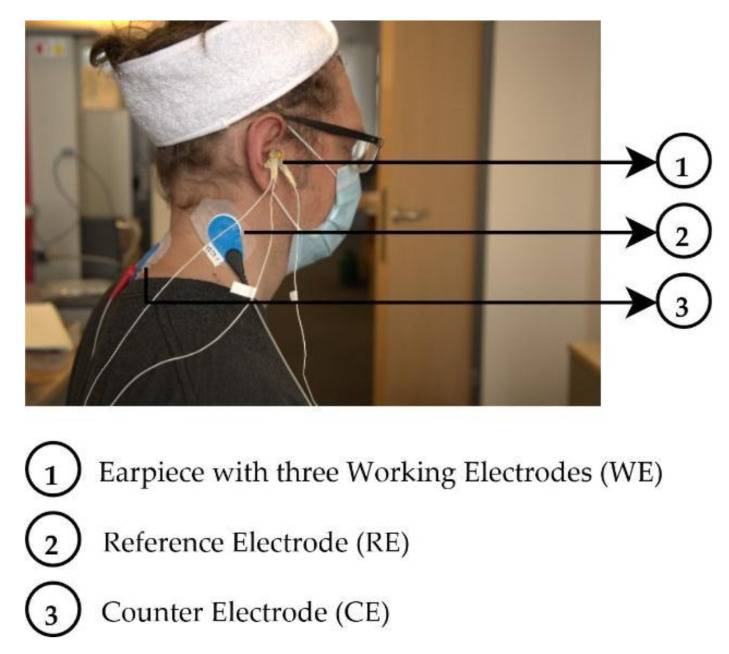
Impedance measurement setup on a subject. Electrodes on earpieces are working electrodes (WE), counter electrode (CE) is at inion, and reference electrode (RE) is between WE and CE, equidistant from both.

**Figure 4:**
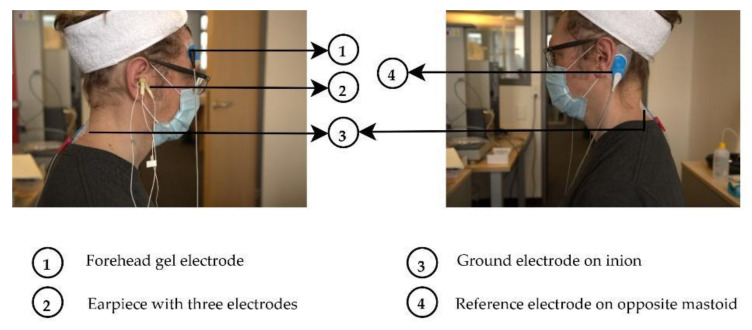
EEG recording setup on a subject. The ground was located at the inion, the reference at the contra-lateral mastoid.

Other than the electrodes attached to the earpieces, all other electrodes utilized for measurements were conductive wet gel electrodes (Ambu^®^ BlueSensor VL (Ballerup, Denmark) [27]. The impedance measurements were performed using a counter electrode at the inion, which is a protruding portion of the occipital bone at the base of the skull (as shown in Figure 3) and the Reference electrode was placed between the inion and the ear, such that it was equidistant from both. Figure 3 and Figure 4 illustrate the electrode placements on the participant under test. The ground electrode was placed at the inion, whilst the reference electrode was placed at the contralateral mastoid for EEG measurements.

### 2.4. Experimental Setup

#### 2.4.1. Experimental Procedure

**Figure 5:**
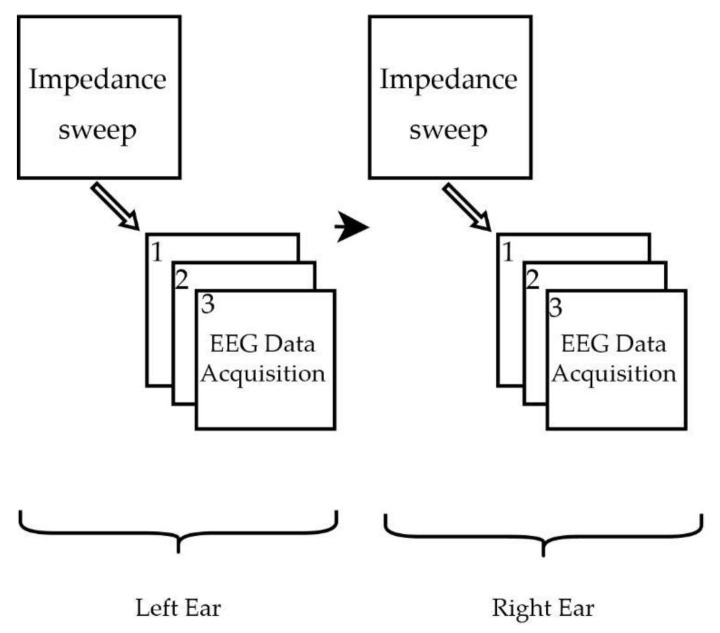
Experimental procedure, the impedance measurements were carried out prior to the EEG recording. Left ear measurements were performed first with electrodes ELE, ELJ, ELH, and Fp1; followed by right ear measurements with electrodes ERE, ERJ, ERH, and Fp2. Each participant was subjected to three trials (designated as EEG Data Acquisition 1, 2 and 3).

Electrode skin contact impedance was measured for each participant before EEG measurement to ensure secure contact to the skin and to avoid cross-channel linking. The electrodes were placed on the subjects as described in Section 2.3. As previously reported in the literature, it has been experimentally observed that ear cerumen increases impedance values in the ear by up to 86% compared to clean ears [28]. Hence, the ear canal was cleaned prior to measurements with alcohol wipes and ear swabs. The ear canal was then dried with swabs before electrode insertion. The measurements were first conducted in the left ear followed by the right ear and each participant’s measurements were collected on the same day. The impedance measurements were conducted initially after 5 min of stabilization time, after the placement of electrodes on the subject, followed by EEG measurement. The session time for EEG measurement consisted of 2 min. For both ears, three trials were conducted for each subject. The experimental procedure is shown in Figure 5.

#### 2.4.2. Electrode Skin Contact Impedance

A frequency response analyzer (PalmSens4) was used to measure the frequency response in a three-wire impedance configuration. The three-wire impedance spectroscopy represents an approximation of the impedance of one electrode along with its interface to the skin and the underlying skin layers. It is most often used in conventional electrochemical applications. The electrode being investigated is referred to as the working electrode (WE), whereas the electrode required to complete the electrical circuit is called the counter electrode (CE). Third electrode, called the reference electrode (RE), is used to determine the potential across the electrochemical interface accurately. All potential measurements in electrochemical systems are defined with respect to a reference electrode, since the absolute potential of a single electrode cannot be measured. Before applying electrodes to the skin, the skin was cleaned with ethyl alcohol to prepare it for the procedure, before the earpiece was compressed and placed into their ears. A drop of saline solution was applied to the electrode before inserting it into the ear to reduce stabilization time. After 5 min of stabilization time, the impedance was measured at 1, 10, 30, and 100 Hz, with the electrode under test functioning as the working electrode (WE), the electrode at the inion acting as the reference electrode (RE), and the electrode between the inion and the ear acting as the counter electrode (CE).

#### 2.4.3. EEG Recording

EEG activity was collected using the OpenBCI Cyton [29]. The low cost, wireless and wearable emphasis of the research is one of the reasons for the use of this device. The OpenBCI hardware has been validated against clinical-grade EEG hardware and is using similar electronics components as most consumer EEG systems [29,30]. The hardware information for the OpenBCI Cyton is described below in Table 2.

An Open Source EEG paradigms library, EEG Notebooks [31] was used to conduct experiments. By combining additional Python scripts with EEG Notebooks, a stimulus presentation software was created. This library was used to handle Bluetooth communication with the OpenBCI Cyton Board, data storage and marker assignments of each stimulus on the raw data. Stimulus presentation and instructions were shown on a computer running on Windows 7.

The presentation software provided visual cues to notify the participants to keep their eyes open for one minute and both visual and auditory cues instructed them to close their eyes for one minute. Auditory and visual stimuli were not provided to evoke responses in the subjects. Cues were provided solely to keep track of timestamps for subsequent signal processing. The subjects were briefed on the experiment beforehand, so the auditory and visual cues served as a time tracker and reminder to keep the behavior of the subjects consistent with one another. The participants were looking at the computer screen and focused their gaze on the center of the computer screen during the eyes-open state. The measurement’s objective was to acquire an EEG recording in different eye states, and not to localize brain activity in response to a stimulus.

### 2.5. Data Analysis

The data analysis for the impedance measurements was carried out using MATLAB 2019a (The Mathworks, Natick, MA, USA) [32]. EEG data pre-processing, analysis, and visualization was conducted using Python. The EEG data pre-processing pipeline made use of prominent scientific computation and visualization python libraries including SciPy, MNE, Pandas, NumPy, Matplotlib, etc. The statistical analysis was performed using IBM SPSS Statistics 28.0.

The impedance measurement data were collected into csv file format for each recording. These files contained the impedance and phase observed at each of the four frequency points. The mean and standard deviation of each electrode was calculated across all of the participants in left and right ear across all frequencies. These results were then plotted for the system’s impedance to evaluate the electrode’s contact and skin-electrode behavior.

**Figure 6:**
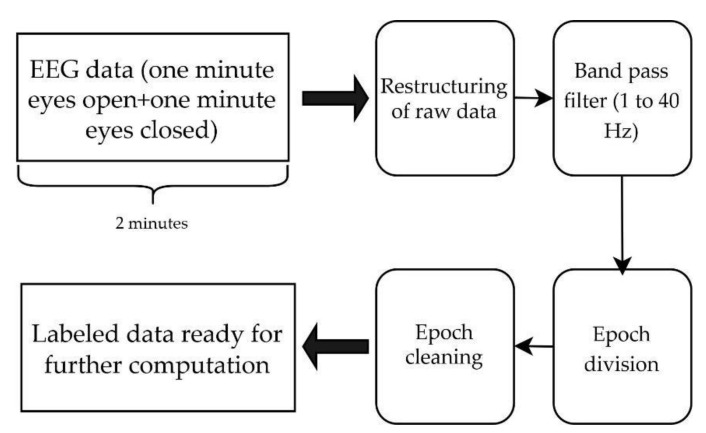
EEG data pre-processing pipeline. EEG data consisted of 2 min data of eyes-open and eyes-closed condition. The raw data were restructured with labels and then bandpass filtered, and split into epochs. Epochs were cleaned according to epoch acceptance criteria to have labeled data which were ready for further computations.

The signal processing and interpretation performed on the recorded data included filtering, transforming, and analyzing the data in order to compare each subject, session, and location statistically. After visually inspecting the data, two subjects showed corrupted data in a few of the ear channels. Few of the channels had flat lines and nothing was recorded; this might have been caused by a loose connection on one of the channels. Data from those subjects were discarded from further investigation. The remaining participants’ data went through the following steps in the pre-processing pipeline (See Figure 6): 1. Data restructuring, 2. bandpass filtering, 3. epoch extraction, 4. epoch cleaning.

1. Restructuring of raw data: First, the data were reorganized to include session-specific information, such as electrode locations corresponding to channels, left/right ear information and stimulation conditions, to make further processing steps more efficient. This produced session-specific information which was stored in unique labels corresponding to the data.
2. Filtering: The labeled restructured continuous raw data were then frequency filtered using a 4th order finite impulse response filter (FIR) with a bandpass of 1 to 40 Hz. The specified bandpass filter level ensured that power line interference and Ac drift is eliminated.
3. The filtered data were then divided into epochs. Each epoch was composed of 5-s windows with a 4-s overlap in frequency analysis to allow for more in-depth investigation (overlapping sliding windows).
4. Epoch cleaning: Each epoch was then cleaned through an automatic artifact rejection function employing different artifact criteria. If an epoch was a simple flat line, it was classified as bad. If the signal amplitude exceeded the threshold of −60 µV to +60 µV, the epoch was classified as an artifact and was labeled as bad. The threshold was chosen through EEG expert manual selection of a threshold for filtered data in 5-s windows. The threshold was determined with the intention to contain a large amount of good data yet to exclude all data with external influence or artifacts.

This investigation did not differentiate between artifact types. The major artifacts originating mainly from eyes blinking, muscle movements, jaw clenching were removed through bandpass filtering and epoch cleaning.

#### 2.5.1. Power Spectral Density

For all acceptable, filtered epochs, the processed data were used to compute a Fast Fourier Transform (FFT) with a Welch transformation. To avoid misleading optimized results owing to many epochs within the same subjects, electrodes, or session combination, the pre-processed data for each channel were averaged over all recordings and participants. The mean and standard deviation results for ear electrodes and forehead channels were plotted in the frequency range of 1 to 40 Hz to investigate the frequency spectrum behavior for all electrode locations in both ears.

The statistical significance of the eyes-open vs eyes-closed conditions was determined using a paired one-tailed t-test with the null hypothesis that the mean alpha and delta of the eyes-open condition PSD are equal to the mean alpha and delta of the eyes-closed condition PSD.

#### 2.5.2. Signal Quality (SNR)

A signal-to-noise ratio (SNR) is a metric used to quantify the contamination level in the frequency and time spectrum of the data and can be used to evaluate the signal quality of the recordings. However, SNR, on the other hand, is an excellent metric for measuring the signal quality of any recorded signal; nevertheless, there is no gold standard for it in continuous EEG recordings. Hence, we estimated SNR in the frequency band using the formula established in [33], which is as follows:

(1) $$\mathrm{SNR}\;=\frac{\mathrm{mean}\left(\mathrm{PSD}\;\mathrm{band}\;\mathrm{of}\;\mathrm{interest}\right)}{\mathrm{mean}\left(\mathrm{PSD}\;\mathrm{signal}\;\mathrm{band}-\mathrm{band}\;\mathrm{of}\;\mathrm{interest}\right)}$$

The band of interest for the alpha paradigm has traditionally been described in the literature to be between 8 and 14 Hz [24]. By implementing the Welch transformation, we were able to establish that the band of interest for alpha during eyes-closed condition was between 7 and 13 Hz, based on the power spectral density plot. Additionally, few other studies also define the alpha band as being between 7 and 13 Hz [34]. The signal power is thus defined as the mean power spectral density in the band of interest (7–13 Hz). Noise power is the mean power outside this band. The ratio of these gives the signal-to-noise ratio.

The signal quality was further investigated by analyzing the results of the artifact rejection. These rejected data were analyzed for patterns across electrode experiments, subjects, channels, and stimuli conditions. This investigation first included analysis of the labels of the affected data, and then looked at the calculated percentage of successful data per context.

#### 2.5.3. Alpha Bandpower Correlation

The absolute alpha bandpower of forehead electrodes (gold-standard) was compared to that of ear electrodes. For this, the processed cleaned data were analyzed to find absolute bandpower in the alpha band (7–13 Hz). The absolute alpha power values of all participants were correlated to investigate the relationship between in-ear electrodes. The Pearson correlation coefficient between in-ear electrodes was evaluated to quantify the similarity of activity detected by ear electrodes. The data examined were not subjected to artifact rejection, which means that both good and bad epochs were considered while analyzing it. The data were compared directly because it was hypothesized that any artifact detected by one of the electrodes in the ear canal would be also detected by the neighboring electrodes in the ear channels.

The same method was applied for the evaluation of the correlation coefficient of the forehead with the in-ear electrodes, as was used for the evaluation of the correlation coefficient of the in-ear electrodes. However, in this instance, often the forehead lacked good epochs for some trial sessions. To maintain consistency between the in-ear and frontal channel data, complete sessions containing bad epochs according to our criteria were discarded in such instances. This discrepancy is usually a common occurrence since the forehead electrode locations are likely to pick up different artifacts and different signal strengths than one would get from the in-ear electrodes. Thus, in the correlation study between the forehead and in-ear channels, only data containing results for all channels were evaluated. This ensured that the number of samples for each channel examined in the correlation remained constant, thus reducing the possibility of a confounding variable.

## 3. Results and Discussion

### 3.1. Electrode Skin Contact Impedance

**Figure 7:**
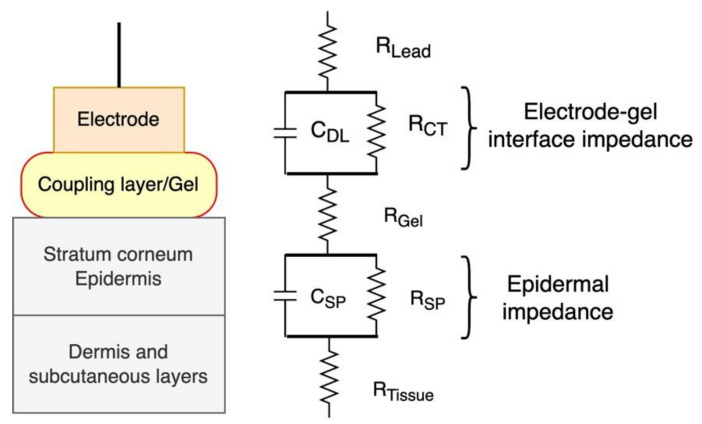
Electrical equivalent circuit of skin electrode interface [35].

A simplified equivalent circuit model of the skin electrode interface is shown in Figure 7 [35]. It comprises the electrode lead resistance, R_Lead_; the electrode-gel interface impedance (a parallel combination of the double-layer capacitance, C_DL_, and the charge transfer resistance, R_CT_); the gel resistance, R_Gel_; the skin impedance (a parallel combination of a capacitance, C_SP_, and a resistance, R_SP_); and the underlying tissue resistance, R_Tissue_. The equivalent circuit model shown here is a simplification of the complex electrical properties of the skin. The outermost skin layer, the stratum corneum, has a high resistance to current transmission. Consequently, the electrode impedance is dominated by the skin resistance. As a result, its dielectric properties and thin thickness, the stratum corneum allows capacitive coupling between a metal electrode placed on the skin’s surface and conductive tissues underneath it. The resistances of the lead, the gel, and the tissue, as well as of the electrode-gel contact is small in comparison to the skin. As a result, skin impedance typically predominates in dry electrode contacts.

**Figure 8:**
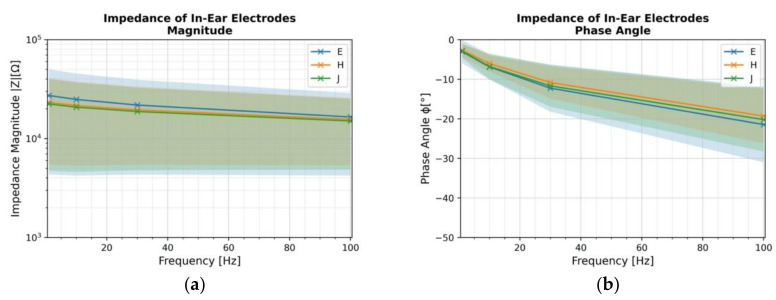
Mean impedance magnitude (**a**) and phase angle (**b**) with standard deviation of electrodes at in-ear locations E, J and H. *n* = 8.

Figure 8 shows the grand average mean impedance magnitude spectra and phase angle with standard deviation of electrodes for all participants in the ear. The measurements in both ears are averaged across participants. Table 3 gives exact values of mean and standard deviations of impedance at 10 Hz. The values range from a minimum of 10 kΩ to a maximum of 92 kΩ at some locations. When comparing three in-ear locations, the values are quite similar but reveal large inter-subject variations.

The quality of biopotential recordings at the skin surface is highly dependent on the electrode-skin interface. Factors that influence the interface include the shape of the electrode and how it conforms to the ear canal. Generic earpieces fit comfortably in the ear canal regardless of the individual’s ear size. If skin electrode contact impedance values are unstable and high, signal quality is reduced resulting in challenging signal acquisition [36]. Through the input impedance of the amplifier, the current noise is converted to voltage noise. Consequently, larger impedances are associated with higher noise levels. Skin thickness and hydration are crucial variables that influence the conductivity of the skin inside the ear canal, as is the effect of sebum and the existence of large apocrine glands [36]. Poor skin conductivity impacts the quality of measurement and is quantified by impedance checks, considering the behavior of skin-electrode impedance is vital before signal acquisition.

As indicated in Table 3, the values for the impedance measurements for all electrodes with a mean of 22.4 kΩ align with the in-ear wet electrode impedance values found in the literature, 34 kΩ (SD = 37 kΩ) [37]. These values also align with the impedance values of cloth electrodes found in the literature, 10 kΩ median with highest impedance on one of the subjects being 19 kΩ [38]. The standard deviation values are significantly higher and are roughly equivalent to the mean values. This high standard deviation indicates that impedance magnitude and phase varies significantly across individuals, which has already been shown in previous research [37]. This may be related to the individual differences in skin properties, including stratum corneum thickness [36]. There is no significant difference in electrode location. This could be attributed to the uniform skin composition in the outer ear canal. Therefore, the placement does not have a significant impact on the signal pickup. In earlier findings [39], for 10 individuals, position E had the lowest impedance, but when the number of subjects is decreased to 8, the location with the lowest impedance value changes, showing once again the significant inter subject variability. The phase plot highlights the capacitive effect of the total impedance which also significantly changes between individuals. The ADS1299 chip used in the Cyton has an input impedance of 1 GΩ. According to [40], a skin impedance of 40 kΩ resulted in no distortion for a 200 MΩ amplifier. The skin contact impedance up to 1 MΩ, for the OpenBCI Cyton, nevertheless, will lead to reliable signal acquisition (<0.1% error). Thus, the impedance values observed are acceptable for EEG data acquisition at all three locations.

### 3.2. Power Spectral Density (PSD)

**Figure 9:**
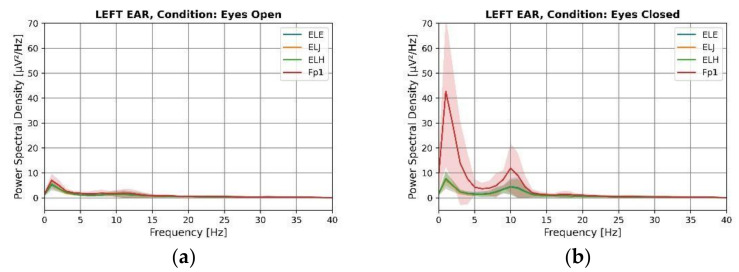
Power spectral density of electrodes for left ear measurements of eyes-open (**a**) and eyes-closed (**b**) conditions.

**Figure 10:**
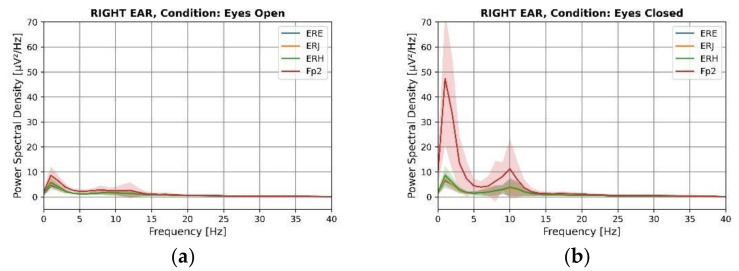
Power spectral density of electrodes for right ear measurements of eyes-open (**a**) and eyes-closed (**b**) conditions.

Figure 9 and Figure 10 display the power spectral density of the data for the left and right ear for eyes-open and eyes-closed conditions averaged across subjects. The alpha modulation can be observed for all electrode locations in both ears during the eyes-closed condition. All electrodes show a clear peak at 10 Hz. Visual inspection of the graph indicates similarities between the forehead and ear locations. The graph shows activity recorded from the forehead with a higher amplitude compared to the ear electrode locations. It has also been observed in the literature that scalp electrodes have a higher amplitude than in-ear electrodes [15], potentially due to an increased electrode size. However, a thicker bone structure between the brain and the surface of the ear canal, as opposed to the bone structure between the brain and the surface of the scalp, may also play an important role [14]. After a visual examination, there is no apparent difference between the different in-ear electrode locations. It is possible that this is because the ear canal is relatively narrow, and all positions are in close proximity in comparison to the distance of the ear canal to the signal sources inside the brain. All ear electrode waveforms closely overlap each other and follow the shape of forehead electrodes. The standard deviation indicates that the amplitude of the alpha peak varies across individuals. Individuals process information differently, using distinct regions of the brain, depending on their cognitive style. Individuals’ mental states during data acquisition may also contribute significantly to alpha generation. As a result, the total height and width of the alpha peak would be expected to vary across different people. The statistical significance tests between the eyes-open and eyes-closed conditions of alpha (Table 4) indicate that the findings are statistically significant across all subjects for both ears (*p* ≤ 0.002).

The Delta power was also significantly increased in the eyes-closed condition compared to the eyes-open condition for the frontal electrodes. Delta activity has been shown to originate in the frontal lobes during the eyes-closed condition [41]. As a result, the large magnitude of delta can be observed when the eyes are closed from frontal electrodes. T. Harmony hypothesized the presence of two distinct types of behavioral inhibition, both of which are reflected by delta waves [42]. ‘Class I inhibition’ refers to the complete deactivation of an excitatory process, as in sleep, resulting in a relaxed, less active state. During the performance of a mental task, ‘Class II inhibition’ would selectively suppress incorrect or irrelevant brain activity. As shown in Figure 8 and Figure 9, the delta waves that were predominantly observed from frontal electrodes represent ‘Class I inhibition’ waves because they appear to be in the eyes-closed condition, suggesting that the subjects were in the relaxed state. Additionally, the delta power of in-ear electrodes was increased, however, not as much as that of forehead electrodes. However, statistical tests revealed this delta inhibition was also recorded by the ear electrodes, ELH and ERH (*p* < 0.05), demonstrating that this delta activation can also be recorded from the ear. The explanation for other in-ear electrodes not recording the same activity could not be determined as all three electrodes exhibit similar impedance. Nevertheless, statistical results demonstrated that in-ear electrodes, like forehead electrodes, can identify delta deactivation even when it is not evident in the graphs.

### 3.3. Signal Quality

**Figure 11:**
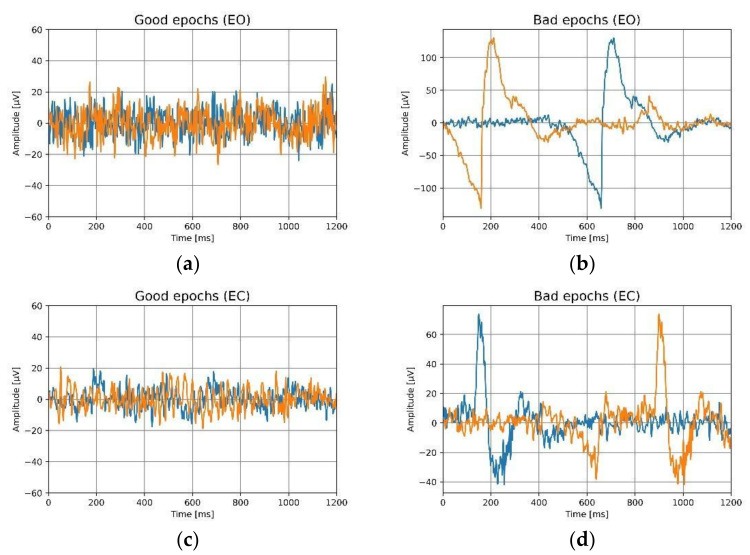
For eyes-open (EO) and the eyes-closed (EC) conditions, the epochs of the ear electrodes are shown. Two distinct epochs are indicated by two distinct colors, (**a**) good epochs for eyes-open, (**b**) bad epochs for eyes-open, (**c**) good epochs for eyes-closed, (**d**) bad epochs for eyes-closed.

**Figure 12:**
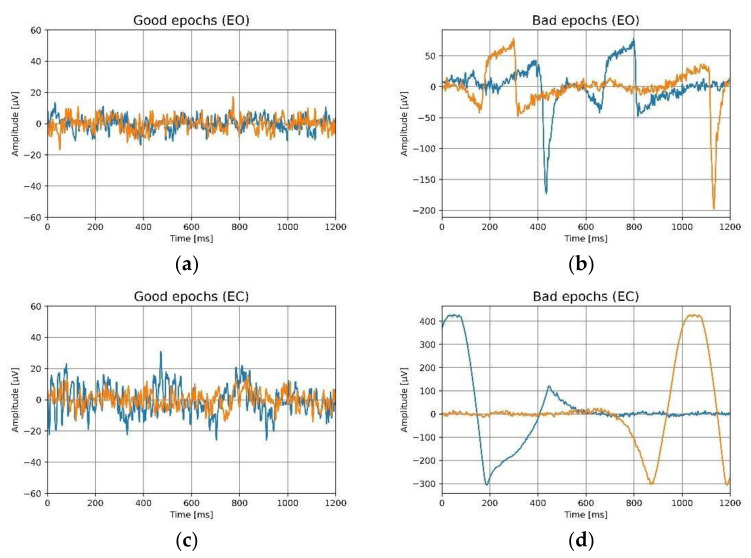
For eyes-open (EO) and the eyes-closed (EC) conditions, the epochs of the forehead electrodes are shown. Two distinct epochs are indicated by two distinct colors, (**a**) good epochs for eyes-open, (**b**) bad epochs for eyes-open, (**c**) good epochs for eyes-closed, (**d**) bad epochs for eyes-closed.

The contaminated epochs, labeled ‘bad’, were removed after the pre-processing steps described in Section 2.5.3. Data quality was assessed by calculating the percentage of good data at each electrode location, and a mean and standard deviation were computed for all trials across all participants. Table 5 summarizes the amount of good data for both ears while the eyes are open and while closed. It can be observed that there is over 98% good data for ear electrodes, irrespective of ear and location in the ear canal. With 46.36% (Fp1, eyes-open) to 78.67% (Fp1, eyes-closed), forehead electrodes have a lower percentage of good data. Furthermore, the percentage of good data is reduced for the eyes-open condition compared to the eyes-closed condition, from 46.36% (Fp1) to 46.52% (Fp2). The investigation into bad data reveals many artifacts on forehead location as there were high amounts of artifacts in the eyes-open condition, which may have been related to the participants’ eye movements, facial muscles, or blinking. Figure 11 and Figure 12 illustrate good and bad epochs for ear and forehead locations. The magnitude of artifacts picked up by the forehead appears to be very high in general, while the magnitude of artifacts picked up by the ears clearly seems relatively small. R. Barry et al. employed Ag/AgCl electrodes and studied an eye blinking artifact on the forehead at the Fp2 position [41]. They observed distinct bursts of activity during blinking. The artifacts they observed resembles the eyes-open artifacts observed in Figure 11 and Figure 12. This implies that, while recording EEG signals, the forehead location is more sensitive to eye movement artifacts compared to in-ear electrodes. Furthermore, the significant standard deviation in the percentage of acceptable data from the frontal electrodes indicates inter-subject variability. This suggest that some participants’ movements were more prominent than others and that artifact contamination is extremely subjective in general.

**Figure 13:**
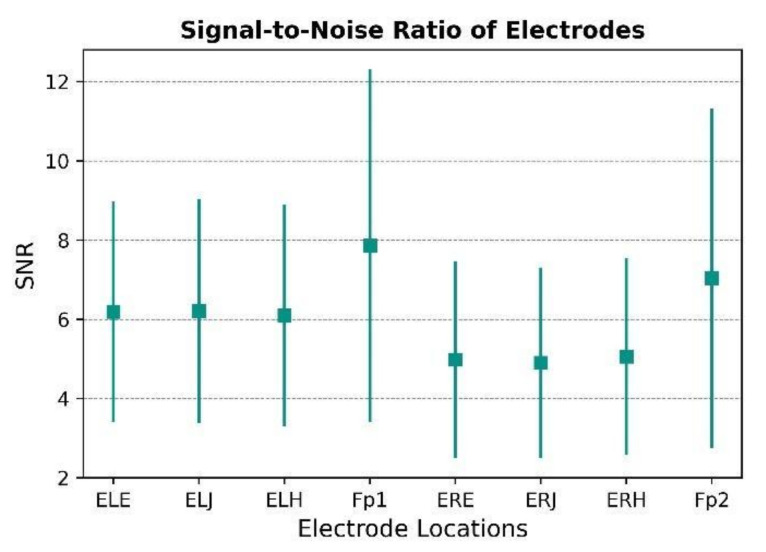
Signal-to-noise ratio (SNR) of electrodes on the left and right sides of the head.

After pre-processing and removing bad epochs, the signal-to-noise ratio was calculated on the filtered data. The signal-to-noise ratio was averaged over all participants and trials. Figure 13 shows the mean and standard deviation of the SNR across each electrode to aid in describing the signal quality. The pre-processing pipeline removed the noise in the signal and improved the SNR. As a result, the values obtained at the frontal location, Fp1, with a mean of 8, and Fp2, with a mean of 7, are rather higher than the values obtained at the in-ear locations. The SNR values at the forehead are comparable to those reported in the literature at the scalp [33]. In-ear electrodes also exhibit a good SNR for EEG data recording, with the lowest value being 5 for the right ear, and the highest value being 6 for the left ear. Since wet gel electrodes cannot be placed in the ear, the SNR values for in-ear must be compared to the dry electrodes, and dry electrodes in the literature have an SNR ranging from 4.3 to 8.9 [33]. Although the SNR is lower for in-ear locations, the variance across individuals is also smaller. Typically, low impedance values signify a low signal to noise ratio; however, there was no correlation observed between the defined signal quality and the skin electrode contact impedance.

### 3.4. Alpha Bandpower Correlation

**Figure 14:**
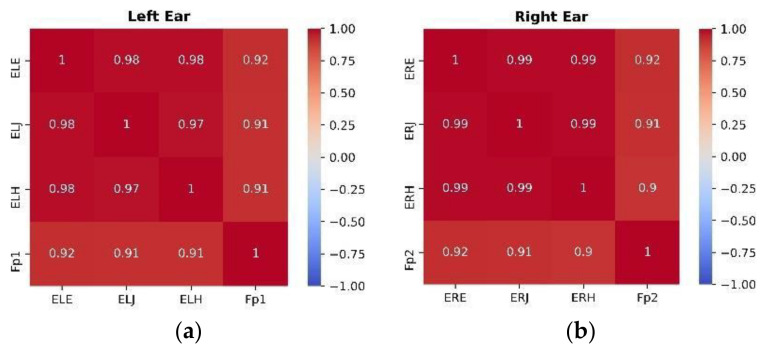
Pearson correlation coefficient matrix of in-ear and forehead locations for left (**a**) and right ear (**b**).

The Pearson correlation coefficient between three in-ear electrodes and frontal electrodes is shown in Figure 14. The in-ear locations are highly correlated with each other, with a correlation coefficient from 0.98 to 0.99 (*p* value = 0). The results are statistically significant. When comparing the PSDs seen in Figure 8 and Figure 9, the alpha activity across in-ear locations is extremely similar since the waveforms overlap with one another. The observed strong correlation indicates a similarity between in-ear locations. Hence, there is no statistically significant difference between the three in-ear locations for alpha activity detection. It may be because the electrodes were near one another and, therefore, the activity detected by them was very similar. The Pearson correlation coefficient between in-ear electrodes and forehead electrodes was also calculated. For both ears, all three in-ear channels show a significant value of correlation of 0.9 to 0.92 with a *p*-value of <0.001. Earlier in-ear locations have been correlated to the T8 position on the scalp, with a high correlation coefficient (0.96) for the alpha and mismatch negativity paradigm [13,42]. In this study, all three in ear locations with high correlation coefficients suggest that the potentials measured at forehead locations and in-ear locations are identical. The forehead and in-ear electrodes share the same reference and the distance between them was small compared to their distance to the reference electrode, so the potentials measured by electrodes seems to be nearly identical. This confirms that the activity measured at in-ear locations is of the same neuronal activity as at the forehead locations, validating in-ear EEG as a comparable and efficient way for measuring brain activity as compared with forehead electrodes.

## 4. Conclusions

The development of ear-EEG systems represents an important step towards ubiquitous EEG that is adapted to the lifestyle of users. Unobtrusive EEG opens up new use cases and application areas in mobile brain imaging. For mobile EEG devices, many designs focus on measuring signals at the forehead since this is a relatively easy to access location on many users as it avoids the complications of using through-hair electrodes on the scalp, which can often present problems with comfort and usability with different hair types and styles across the world. The current work investigated the performance between forehead and ear canal located electrodes to establish the differences in performance, but also to establish the feasibility of creating universal fit electrodes located in the ear canal for mobile BCI design.

The current work draws a strong comparison to previous research in the in-ear EEG field. Previously, D. Looney et al. demonstrated a correlation coefficient of 0.95, 0.83 between the in-ear and mastoid (M1) positions and between the in-ear and T7 positions when using in-ear Ag/AgCl electrodes for the alpha attenuation study [11]. Between in-ear positions, the highest correlation coefficient of 0.99 was observed. This is consistent with the results obtained with electrodes in the current study. Mikkelsen et al. established that the ratio of alpha power between closed and open eyes for in-ear electrodes is around 1.5. Visual examination of alpha powers indicates that the ratio for alpha power will be between 1.5 and 2 for the current in-ear electrode results [10]. Kappel et al. showed successful dry in-ear alpha recordings referenced to a Cz position [21]. While these investigations all confirmed the successful recording of in-ear electroencephalograms, there is no information on their comparison to forehead locations. Our investigation established a correlation coefficient of 0.92 of in-ear locations with the forehead.

It was shown that the difference in motion artifacts related to eye movement study can be reduced with ear EEG, as compared to the forehead. This suggests that a BCI system designed based on in-ear EEG could capture more stable brain activity data than using forehead located electrodes. Although forehead EEG devices have been around for many years, they have not seen wide adoption. In-ear EEG ideally has a place in neuroimaging alongside high resolution full-scalp EEG systems. Full-scalp systems are ideally suited to clinics, hospitals, and laboratories, while ear EEG systems may be used in everyday life to capture brain activity at a lower signal spatial resolution in the brain, but over longer periods of time. Forehead systems may be advantageous for some applications such as sleep, or when integrated into helmets (sports, space, hazardous applications). If integrated into earbud headphones, however, in-ear EEG may hold great promise as a candidate to enable “every day” BCI designs.

Validating alpha activity in the ear with a universal earpiece system versus the forehead is important as there are many applications in wellness, and potentially in other areas, where ubiquitous brain imaging measured over the daily life of a person may provide insights not obtainable with other systems that cannot integrate as discreetly in society (such as forehead-based electrode systems). Future work will build upon these results to look at different frequency bands and frequency response characterization such as delta, theta, and gamma activity. The reliable classification of EEG at the ear may also lead to the investigation of sleep micro-architectures, such as k-complexes and spindles, that may offer new ways to characterize sleep behavior.

Some of the limitations of this research were as follows. One of these being the paradigm used was only eyes-open and eyes-closed alpha. Future research may expand on a variety of other paradigms, including mismatch negativity (MMN) and, more importantly, the Auditory steady state response (ASSR), which is utilized with in-ear electrodes. In addition to adopting various other paradigms, the population size can also be expanded.

Future work will build upon these results to look at different frequency bands and frequency response characterization, such as theta and gamma activity. The reliable classification of EEG at the ear may also lead to the investigation of sleep micro-architectures, such as k-complexes and spindles, which will also offer areas to expand into sleep characterization. The use of electrodes based on expanded foam and conductive fabric provided a good system to investigate electrode placement as well as performance. The next step in this body of work is to establish a material system which provides a stable dry interface for electrode performance and conformability, as well as a path towards industrialization. This can support the scaling of the ear EEG electrode technologies and provide more ways to integrate brain computer interface designs into consumer devices, such as earbuds and headphone designs.

## Figures and Tables

**Table 1:** State of the art consumer devices which use head and forehead channels for EEG measurements.

Device	Electrode Type	Electrode Locations	Applications
InteraXon Muse	Metal	4 channels, AF7, AF8, TP9, TP10	Sleep tracking and meditation
Macrotellect BrainLink	Metal	3 channels, FP1, FPz, FP2	Meditation
Neurosky Mindwave	Metal	1 channel, AFz	Attention, meditation
Emotiv Insight	Semi-dry polymer electrodes	5 channels, AF3, AF4, T7, T8, Pz	Meditation and research
BrainCo FocusCalm	Metal	3 channels, AF7, AF8, FPz	Meditation

**Table 2:** Specifications of OpenBCI Cyton board.

Parameters	Values
CMRR	110 dB
DC input impedance	500 MΩ
Sampling rate ADC resolution	250 Hz 24 bits

**Table 3:** Magnitude of mean and standard deviation of impedance of the in-ear electrodes at 10 Hz.

	Mean (kΩ)	Standard Deviation (kΩ)
E	24.9	20.6
J	20.8	16.1
H	21.6	16.3
Mean	22.4	17.7

**Table 4:** *t* test statistics of electrodes in the left and right ear between eyes-open and eyes-closed conditions.

	Delta (δ)		Alpha (α)	
	T Value	*p* Value	T Value	*p* Value
ELE	1.656	0.710	3.652	0.004
ELJ	1.612	0.750	4.233	0.002
ELH	2.945	0.011	3.897	0.003
Fp1	3.314	0.006	4.061	0.002
ERE	1.876	0.051	3.208	0.007
ERJ	1.655	0.071	3.267	0.007
ERH	2.073	0.038	3.271	0.007
Fp2	3.046	0.009	4.159	0.002

**Table 5:** Amount of good data for left and right ear in eyes-open and eyes-closed conditions.

	Left Ear			Right Ear		
	Locations	Mean (%)	Standard Deviation	Locations	Mean (%)	Standard Deviation
**Eyes Open**	ELE	98.45	1.46	ERE	99.47	1.10
	ELJ	98.56	2.12	ERJ	99.17	1.55
	ELH	99.13	1.37	ERH	98.79	1.68
	Fp1	46.36	40.73	Fp2	46.52	42.19
**Eyes Closed**	ELE	99.62	1.07	ERE	99.62	1.07
	ELJ	99.05	1.80	ERJ	100	0
	ELH	99.62	1.07	ERH	99.09	1.71
	Fp1	78.67	24.83	Fp2	77.05	27.20

## Data Availability

The dataset supporting the conclusions of this article is not available due to privacy and ethical reasons.
